# Biochar as a protective adsorbent against cadmium and lead stress in parsley (*Petroselinum crispum*): a study on growth, nutrient uptake, and antioxidant enzyme activities

**DOI:** 10.7717/peerj.21144

**Published:** 2026-04-08

**Authors:** Handan Sarac

**Affiliations:** Sivas Vocational School of Technical Sciences, Sivas Cumhuriyet University, Sivas, Türkiye

**Keywords:** Biochar, Food safety, Heavy metal phytotoxicity, Antioxidant defense mechanism

## Abstract

**Background:**

Heavy metal contamination, particularly from cadmium (Cd) and lead (Pb), poses serious threats to plant growth, nutrient uptake, antioxidant defense mechanisms, and food safety. Biochar (BC) is known to improve soil quality and reduce heavy metal toxicity in plants. This study aimed to assess the impacts of Cd and Pb stress on nutrient element concentrations and antioxidant enzyme activities in parsley (*Petroselinum crispum*), and to evaluate the extent to which BC mitigates these detrimental effects.

**Methods:**

The study was conducted in 3 kg capacity pots arranged in a randomized factorial design with three replications. Parsley (*P. crispum*) was chosen as the test plant. Cd and Pb were applied separately as CdSO_4_ and Pb(NO_3_)_2_ at concentrations of 0, 2.5, and 5 mg kg^−1^. Biochar was incorporated into the soil at 0% and 1% (w/w) rates. Dry weight, heavy metal accumulation, nutrient element concentrations, and antioxidant enzyme activities—catalase (CAT), peroxidase (POD), and ascorbate peroxidase (APX)—were measured to determine the physiological and biochemical responses of plants to heavy metal stress and BC application.

**Results:**

The results showed that the highest dry weight (11.05 g pot^−1^) was observed in the control group with 1% BC application, representing a 17.8% increase compared to the non-BC application. This increase in dry weight was found to be statistically significant. While the BC application reduced Cd concentration in the plant by 14.4%, it had no significant effect on Pb concentration. Under heavy metal stress, BC improved nutrient acquisition, elevating potassium (K), magnesium (Mg), iron (Fe), zinc (Zn), and manganese (Mn) concentrations by 15.9%, 5.9%, 69.3%, 5.5%, and 17.6%, respectively. Exposure to Cd and Pb inhibited CAT, POD, and APX activities; however, BC application alleviated this suppression, increasing enzyme activities by 20.4%, 66.7%, and 25.0%, respectively.

**Conclusions:**

BC application increased dry weight, nutrient uptake, and antioxidant enzyme activities in parsley plants exposed to Cd and Pb stress. Overall, the findings indicate that BC enhances the growth performance and antioxidant defense capacity of parsley plants grown in Cd- and Pb-contaminated soils, highlighting its strong potential as a sustainable soil amendment for mitigating heavy metal toxicity and safeguarding crop productivity in contaminated agricultural systems.

## Introduction

As a result of technological and economic development, climate change, overexploitation of natural resources, and rapid urbanization, the environment has been increasingly exposed to various toxins and pollutants. Among these environmental toxins, heavy metals have been recognized as one of the primary contributors to environmental pollution, which has become a major global concern in recent years (Sheydaei et al., 2022; Aybar, Bilgin & Sağlam, 2015). The sources of heavy metal pollution include both natural processes and anthropogenic activities. Natural processes are mainly associated with the weathering of parent rocks and soil formation (Kohzadi et al., 2019). However, anthropogenic contributions—such as agricultural practices involving organic and mineral fertilizers, pesticide application, and the use of wastewater, as well as industrial activities- result in the release of heavy metals into the environment due to human intervention (Ulusu, Öztürk & Elmastaş, 2017; Kavvadias et al., 2012; Ak & Yücel, 2011). Soil contaminated due to heavy metal accumulation exhibits reduced bioavailability, adversely affecting plant growth and ecosystem functioning (Algan & Bilen, 2005). While certain heavy metals—including iron (Fe), copper (Cu), nickel (Ni), manganese (Mn), cobalt (Co), chromium (Cr), and zinc (Zn)—are essential micronutrients required in trace amounts for normal plant growth and metabolism, others, such as arsenic (As), cadmium (Cd), and lead (Pb), have no known physiological function in plants. These non-essential metals are considered toxic even at low concentrations and are detrimental to plant development (Arsenov et al., 2021; Akgül et al., 2016; Rascio & Navari-Izzo, 2011). A common mechanism underlying heavy metal toxicity is the induction of oxidative stress. Heavy metals disrupt electron transport processes, particularly in chloroplast membranes, leading to increased production of reactive oxygen species (ROS) (Rascio & Navari-Izzo, 2011). When oxidative stress becomes severe, the antioxidant defense system may be insufficient, resulting in irreversible damage to plant tissues (İriş & Çınar, 2019). Plants possess both enzymatic and non-enzymatic antioxidant defense mechanisms to mitigate the harmful effects of ROS. Non-enzymatic antioxidants include lipid-soluble components (such as alpha-tocopherol and β-carotene) and water-soluble components (such as glutathione and ascorbate), whereas the main enzymatic antioxidants comprise ascorbate peroxidase (APX), superoxide dismutase (SOD), catalase (CAT), and glutathione reductase (GR) (Mishra et al., 2023). At low concentrations, heavy metals such as Cd, Cr⁶^+^, mercury (Hg), Ni, Pb, and Fe generally induce an increase in the activities of antioxidant enzymes such as peroxidase (POD), SOD, and CAT. However, as the concentration of heavy metals increases, the activities of these enzymes tend to decline (Cheng, 2003).

High concentrations of heavy metal phytotoxicity (poisonous effects on plants) cause stress in plants, leading to inhibited growth and development, disruption of enzymatic functions, root damage, impairment of storage processes, reduced photosynthetic activity, decreased nutrient uptake, and ultimately, significant yield losses (Yağdı, Kaçar & Azkan, 2000). Therefore, heavy metal contamination is considered a major factor adversely affecting crop production yield and quality parameters (Ak & Yücel, 2011). Furthermore, consuming plant-based foods contaminated with heavy metals poses serious health risks to humans, representing the final link in the food chain (Mirmohammadmakki et al., 2023).

Cd, a non-essential and toxic element for living organisms, is widely released into the environment due to its extensive use in various industrial sectors such as mining, metal smelting, electroplating, and producing paints and pigments (Ulusu, Öztürk & Elmastaş, 2017). The primary sources of Cd input into agricultural soils include the application of phosphate fertilizers, soil amendments using municipal sewage sludge, and atmospheric deposition (Kavvadias et al., 2012). Due to its long biological half-life, Cd persists in the soil for extended periods and readily accumulates in plant-derived food products (Zuhra et al., 2024). Owing to its high mobility and water solubility, Cd can be easily absorbed by plant roots and subsequently translocated to aerial parts, where it tends to accumulate in the leaves (Piekut et al., 2018). Cd has toxic effects on plants, negatively influencing physiological and biochemical processes such as growth, photosynthesis, and nutrient uptake (Asati, Pichhode & Nikhil, 2016). Moreover, it induces oxidative damage in plant cells by promoting the formation of free radicals and ROS (Wang et al., 2022).

Pb, on the other hand, is a highly toxic element that is widely present in the ecosystem and causes serious environmental problems, particularly in soil–plant systems (Rahman et al., 2024). The toxic effects of Pb vary depending on its concentration, chemical form (salt type), soil properties, and the plant species or variety. Harmful levels of Pb interfere with metal ions bound to functional groups within macromolecules, disrupt photosynthesis, alter mineral nutrition, and affect the activity of enzymes in regulating the plant’s water status. These disruptions negatively impact key physiological processes such as seed germination, shoot development, tolerance index, and root and shoot dry biomass (Ak & Yücel, 2011).

Biochar (BC) is a product derived from the pyrolysis of biomass under low-oxygen conditions (Lehmann & Joseph, 2024). Incorporating BC into soil has attracted increasing attention for two primary reasons. First, due to its high chemical stability, BC is considered an ideal material for long-term carbon sequestration in soil (Woolf et al., 2010). Second, BC enhances soil fertility by improving nutrient retention capacity, thereby promoting plant growth. Additionally, it is regarded as an effective soil amendment owing to its positive effects on soil structure and biological properties (Uzoma et al., 2011; Laird et al., 2010). Several studies have highlighted the growing interest in BC due to its wide range of applications, including improving soil fertility, enhancing crop productivity, increasing soil carbon stocks, and remediating environmental pollutants (Haris et al., 2021). At the end of a study in which 0, 5, 10, 15, and 20 t ha^−1^ BC derived from hazelnut shell and 5, 10, and 20 t ha^−1^ mature animal manure were applied to a soil cultivated with tomato plants, some chemical soil properties (organic matter, total nitrogen (N), available phosphorus (P) and potassium (K)) and several soil enzymes (dehydrogenase, urease, arylsulfatase, and alkaline phosphatase) were determined. The results indicated that BC and animal manure applications increased soil dehydrogenase, urease, and arylsulfatase enzyme activities, and these increases were statistically significant (*P* < 0.01). In addition, BC and animal manure applications enhanced soil organic matter content as well as available macronutrients such as nitrogen (N), phosphorus (P), and K (Yilmaz & Ergun, 2019). BC derived from waste materials is considered a cost-effective adsorbent for the remediation of soils contaminated with heavy metals (Chen et al., 2019). BC has attracted attention as a renewable and efficient adsorbent that can be produced from a wide range of materials such as plant stems, corn cobs, poultry manure, plant leaves, coconut shells, animal manure, and rice husks (Luo et al., 2019). Heavy metals such as As, Cd, Pb, Cr, and Hg can be retained by BC in soil or water through mechanisms including ion exchange, complexation with free functional groups, surface precipitation, and physical adsorption (Li et al., 2017).

Parsley (*P. crispum*), a widely cultivated plant species worldwide, belongs to the Apiaceae family and is native to the Mediterranean region (Mirmohammadmakki et al., 2023; Sheydaei et al., 2022). Its aromatic leaves, which are rich in various vitamins and minerals, are commonly used in culinary applications; in addition, due to its phenolic compounds and flavonoids, parsley also has recognized applications in modern medicine (Mirmohammadmakki et al., 2023). Parsley exhibits a wide range of biological properties, including hypoglycemic, diuretic, antihypertensive, and aorta-protective effects, as well as notable antioxidant and antibacterial activities (Arsenov et al., 2021; Bibi et al., 2016). In Turkey, parsley is widely consumed and can grow easily in open fields, even in areas contaminated with heavy metals (Ulusu, Öztürk & Elmastaş, 2017). Literature data indicate that many medicinal plants, including parsley, have the ability to accumulate heavy metals in their edible parts (Kohzadi et al., 2019; Bibi et al., 2016). In this context, the capacity of plants to accumulate heavy metals becomes a significant drawback when it comes to edible plant species (Arsenov et al., 2021).

In this study, the potential contamination in parsley (*P. crispum*) plants exposed to different concentrations of Cd and Pb was investigated, with a focus on the effects of these heavy metals on nutrient element concentrations and antioxidant enzyme activities, as well as the effects of BC application on these factors. The study hypothesizes that BC application enhances nutrient uptake and antioxidant enzyme activities in parsley plants grown under Cd and Pb stress.

## Materials and Methods

### Plant growth conditions and Cd and Pb applications

In this study, soil samples were collected from the experimental field of Sivas Cumhuriyet University at 0–20 cm depth and passed through a 2 mm sieve (Table 1). The soil was slightly alkaline, low in organic matter, calcareous, and clay-loam textured, with low phosphorus and high potassium concentrations. The study was conducted under controlled greenhouse conditions using a completely randomized factorial design with three replications. Plastic pots with a capacity of 3 kg were used. Parsley (*P. crispum*) was selected as the test plant species. Certified parsley (*Petroselinum crispum* L.) seeds (Italian Giant cultivar) used in this study were obtained from Golden Seed (İzmir, Türkiye). This species is a widely cultivated broad-leaf variety and is not listed as endangered or protected under the Convention on International Trade in Endangered Species of Wild Fauna and Flora (CITES). All experimental procedures were conducted in accordance with institutional, national, and international guidelines for plant research.

**Table 1:** Some properties of the soil and biochar.

Properties	Unit	Soil	Biochar
**pH**	(1:1 H_2_O)	7.52	8.4
**EC**	dS m^−1^	0.21	4.07
**Surface area**	m^2^ g^−1^	–	224
**Texture**		CL	–
**CaCO**_**3**_	%	13.7	–
**Org. matter**	%	1.91	–
**Salt**	%	0.02	–
**C**	%	–	44.6
**N (Total)**	%	–	0.57
**P**	g kg^−1^	0.012	2.6
**K**	g kg^−1^	0.415	3.4
**Zn**	mg kg^−1^	0.47	19
**Pb**	mg kg^−1^	1.17	0.82
**Cd**	mg kg^−1^	0.10	0.53
**Fe**	mg kg^−1^	2.05	325
**Cu**	mg kg^−1^	0.79	1.7
**Mn**	mg kg^−1^	1.14	73

As a base fertilization, each pot received 200 mg N kg^−1^ (as Ca(NO_3_)_2_ $\cdot$ 4H_2_O), 100 mg P kg^−1^ and 125 mg K kg^−1^ (as KH_2_PO_4_), and 2.5 mg Zn kg^−1^ (as ZnSO_4_·7H_2_O) at the time of sowing.

The BC used in the experiment was produced by slow pyrolysis of peanut shells, a widely cultivated crop residue in the Mediterranean region, at 500 °C for 4 h (Table 1), and applied to the soil at 0% and 1% (w/w) rates. In the study, the 1% BC rate was determined based on previous studies. Cd and Pb were used at concentrations of 0, 2.5, and 5 mg kg^−1^ in the forms of CdSO_4_ and Pb(NO_3_)_2_, respectively. Cd and Pb were not applied to the plants used as the control group to allow for comparisons. Cd and Pb applications were applied to the soil as a single application at sowing. In the study, parsley plants were regularly irrigated with distilled water.

### Determination of nutrient element and heavy metal concentrations

Approximately 50 days after sowing, the plants were harvested by cutting them about 1 cm above the soil surface and brought to the laboratory. The plant samples were washed with tap water, followed by 0.1 N HCl solution, then rinsed with deionized water. Leaf samples were placed individually into article bags and dried in a forced-air oven at 70 °C until reaching constant weight. The samples that reached constant weight were weighed in grams using a precision balance to determine dry weight. The dried samples were then ground using a plant grinding mill. A 0.2 g portion of ground plant material was weighed and burned in a microwave device (Milestone Ethos Easy Advanced Microwave Digestion System model; Milestone, Sorisole, Italy) in a H_2_O_2_ and HNO_3_ (2 mL of 35% H_2_O_2_ and 5 mL of 65% HNO_3_) acid mixture according to the wet combustion method. The final volume was adjusted to 20 mL with deionized water, and the solution was filtered through blue-band filter paper.

P concentration was determined colorimetrically using a spectrophotometer at 882 nm according to the method of Murphy & Riley (1962). The concentrations of Cd, Pb, K, calcium (Ca), Mg, Zn, Mn, Fe, and Cu were measured using an Atomic Absorption Spectrophotometer (Shimadzu AA-7000), according to the method described by Kacar & Inal (2008). N content was determined using the Kjeldahl distillation method (Bremner, 1965).

### Antioxidant enzyme analysis

To determine antioxidant enzyme activity, 0.3 g of fresh parsley leaves (from control and treated plants) were ground in a porcelain mortar with liquid nitrogen until a fine powder was obtained. The powder was then homogenized in 50 mM KH_2_PO_4_ buffer (pH 7.0). The resulting homogenate was centrifuged at 15,000 rpm for 20 min at 4 °C. The supernatants obtained after centrifugation were used to determine the activities of the antioxidant enzymes CAT, POD, and APX.

CAT activity was determined spectrophotometrically at 240 nm by following the protocol described by Ulusu, Öztürk & Elmastaş (2017). One unit of enzyme activity was defined as the amount of enzyme required to decompose 1 μmol of H_2_O_2_ per minute, using the extinction coefficient for H_2_O_2_ (0.036 cm^2^ μmol^−1^).

POD activity was determined spectrophotometrically at 470 nm based on the oxidation of guaiacol, following the method described by Angelini, Manes & Federico (1990). The extinction coefficient of tetraguaiacol (26.6 mM^−1^ cm^−1^) was used for the calculation of POD activity.

APX activity was determined spectrophotometrically at 290 nm according to the method described by Wang, Jiao & Faust (1991). The activity was calculated using the extinction coefficient of ascorbate (2.8 mM^−1^ cm^−1^). The enzyme activity results were expressed as enzyme units (EU) per gram of leaf tissue (EU g^−1^ fw).

### Statistical analyses

The effects of different concentrations of Cd and Pb on nutrient element concentrations and antioxidant enzyme activities (CAT, POD, and APX) in parsley plants and the effect of BC application were analyzed using two-way analysis of variance (ANOVA). When significant differences were observed (*P* < 0.05), Tukey’s test was applied. All statistical analyses were conducted using IBM SPSS Statistics, version 22.

## Results

**Figure 1:**
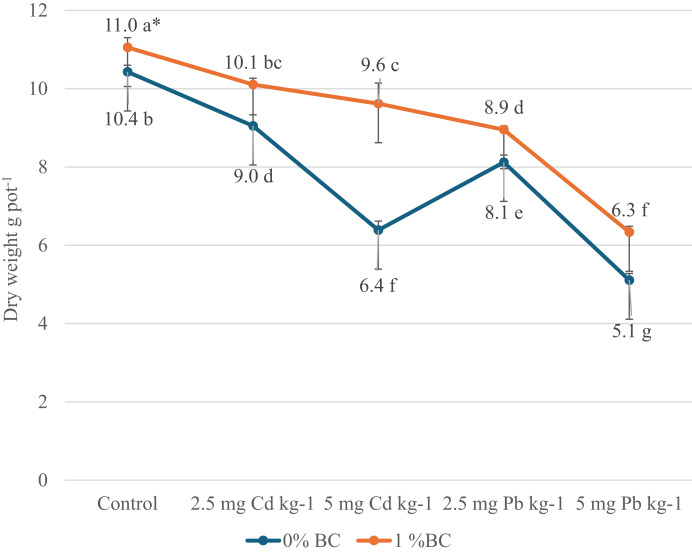
Effects of different doses of BC, Cd, and Pb applications on dry weight of parsley plants (g pot^−1^). *The Cd and Pb data were subjected to separate statistical analyses.

**Figure 2:**
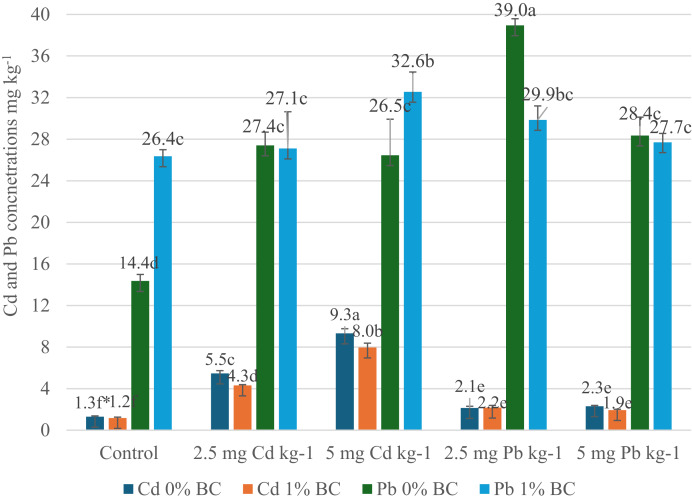
Effects of different doses of BC, Cd, and Pb applications on Cd and Pb concentrations of parsley plants (mg kg^−1^). *The Cd and Pb data were subjected to separate statistical analyses.

The effects of different doses of BC, Cd, and Pb applications on the dry weight of parsley plants are presented in Fig. 1, their effects on Cd and Pb concentrations are shown in Fig. 2, effects on N, P, and K concentrations are provided in Table 2, effects on Ca and Mg concentrations are given in Table 3, and effects on Fe, Zn, Mn, and Cu concentrations are presented in Table 4.

**Table 2:** Effects of different doses of BC, Cd, and Pb applications on N, P, and K concentrations of parsley plants (%).

Applications		N	P	K
		(%)		
0% BC	Control	1.71 ± 0.04^e*^	0.13 ± 0.01^f^	3.61 ± 0.11^c^
	2.5 mg Cd kg^−1^	3.21 ± 0.06^c^	0.15 ± 0.00^de^	5.02 ± 0.13^ab^
	5 mg Cd kg^−1^	3.58 ± 0.08^ab^	0.14 ± 0.01^e^	4.91 ± 0.55^b^
	2.5 mg Pb kg^−1^	3.90 ± 0.42^a^	0.30 ± 0.01^a^	3.62 ± 0.05^c^
	5 mg Pb kg^−1^	3.38 ± 0.11^bc^	0.16 ± 0.00^cd^	5.16 ± 0.04^ab^
**Average**		**3.15 A**	**0.18 A**	**4.46 B**
1% BC	Control	3.15 ± 0.07^c^	0.19 ± 0.00^b^	4.93 ± 0.07^ab^
	2.5 mg Cd kg^−1^	1.24 ± 0.48^e^	0.12 ± 0.01^f^	5.09 ± 0.01^ab^
	5 mg Cd kg^−1^	2.60 ± 0.28^d^	0.12 ± 0.01^f^	5.35 ± 0.08^a^
	2.5 mg Pb kg^−1^	3.59 ± 0.04^ab^	0.17 ± 0.01^c^	5.25 ± 0.33^ab^
	5 mg Pb kg^−1^	2.49 ± 0.06^d^	0.17 ± 0.00^c^	5.23 ± 0.02^ab^
**Average**		**2.61 B**	**0.15 B**	**5.17 A**

**Notes:**

*P* < 0.05.

Mean of three replicates and ± is a standard error.

*The letters used in the table indicate the statistical differences between the groups.

**Table 3:** Effects of different doses of BC, Cd, and Pb applications on Ca and Mg concentrations of parsley plants (%).

Applications		Ca	Mg
		(%)	
0% BC	Control	1.39 ± 0.05^c^*	0.93 ± 0.02^ab^
	2.5 mg Cd kg^−1^	1.71 ± 0.25^bc^	0.83 ± 0.01^d^
	5 mg Cd kg^−1^	1.91 ± 0.02^ab^	0.91 ± 0.04^bc^
	2.5 mg Pb kg^−1^	2.09 ± 0.05^a^	0.84 ± 0.03^d^
	5 mg Pb kg^−1^	2.09 ± 0.11^a^	0.75 ± 0.01^e^
**Average**		**1.84 A**	**0.85 B**
1% BC	Control	2.12 ± 0.16^a^	0.87 ± 0.02^cd^
	2.5 mg Cd kg^−1^	1.69 ± 0.23^bc^	0.87 ± 0.03^cd^
	5 mg Cd kg^−1^	1.49 ± 0.40^c^	0.91 ± 0.03^bc^
	2.5 mg Pb kg^−1^	1.61 ± 0.08^bc^	0.96 ± 0.01^a^
	5 mg Pb kg^−1^	2.24 ± 0.02^a^	0.87 ± 0.01^cd^
**Average**		**1.83 A**	**0.90 A**

**Notes:**

*P* < 0.05.

Mean of three replicates and ± is a standard error.

*The letters used in the table indicate the statistical differences between the groups.

**Table 4:** Effects of different doses of BC, Cd, and Pb applications on Fe, Zn, Mn, and Cu concentrations of parsley plants (mg kg^−1^).

Applications		Fe	Zn	Mn	Cu
		mg kg^−1^			
0% BC	Control	270.4 ± 12.9^e^*	31.5 ± 2.8^f^	137.4 ± 5.5^c^	9.2 ± 1.1^c-d^
	2.5 mg Cd kg^−1^	348.9 ± 2.8^d^	45.4 ± 0.4^cd^	117.3 ± 2.3^d^	11.2 ± 0.1^b^
	5 mg Cd kg^−1^	273.1 ± 26.3^e^	39.0 ± 8.8^de^	90.3 ± 2.1^e^	7.8 ± 0.3^ef^
	2.5 mg Pb kg^−1^	124.7 ± 2.5^h^	80.6 ± 2.6^a^	139.2 ± 7.7^c^	17.8 ± 0.6^a^
	5 mg Pb kg^−1^	216.8 ± 12.2^f^	41.8 ± 1.3^de^	113.8 ± 7.6^d^	9.4 ± 0.4^bc^
**Average**		**243.8 B**	**47.6 B**	**119.6 B**	**11.1 A**
1% BC	Control	674.6 ± 22.2^a^	50.9 ± 2.6^c^	147.5 ± 7.6^c^	10.2 ± 0.2^bc^
	2.5 mg Cd kg^−1^	166.0 ± 6.7^g^	42.3 ± 0.9^de^	106.3 ± 2.9^d^	7.4 ± 0.1^f^
	5 mg Cd kg^−1^	144.1 ± 22.1^gh^	36.5 ± 1.0^ef^	85.4 ± 6.4^e^	5.3 ± 0.8^g^
	2.5 mg Pb kg^−1^	559.2 ± 8.4^b^	61.6 ± 3.0^b^	173.3 ± 6.4^b^	9.3 ± 1.6^bc^
	5 mg Pb kg^−1^	520.2 ± 28.8^c^	59.9 ± 4.6^b^	190.8 ± 3.5^a^	8.3 ± 0.1^d–f^
**Average**		**412.8 A**	**50.2 A**	**140.6 A**	**8.1 B**

**Notes:**

*P* < 0.05.

Mean of three replicates and ± is a standard error.

*The letters used in the table indicate the statistical differences between the groups.

In the study, both 0% and 1% BC applications reduced the dry weight of the parsley plant in all heavy metal applications. However, it was determined that the dry weight of the parsley plant was higher with the 1% BC application compared to the 0% BC application (Fig. 1). The highest dry weight was determined in the control group with 11.05 g pot^−1^ under the 1% BC application. This was followed by the control group under the 0% BC application with 10.43 g pot^−1^. In the study, the lowest dry weight was observed in the 5 mg Pb kg^−1^ application under both 0% and 1% BC applications (5.11 and 6.33 g pot^−1^, respectively). When the average dry weight was evaluated, the 1% BC application increased the dry weight of parsley plants by 17.8% compared to the 0% BC application. In the study, the highest difference in dry weight was observed in the 5 mg Cd kg^−1^ application. While a yield of 6.39 g pot^−1^ was obtained under the 0% BC application, a yield of 9.62 g pot^−1^ was achieved under the 1% BC application, resulting in a 50.5% increase. Similar increases were observed as follows: 5.99% in the control application, 11.66% in the 2.5 mg Cd kg^−1^ application, 10.3% in the 2.5 mg Pb kg^−1^ application, and 23.9% in the 5 mg Pb kg^−1^ application.

When evaluating Fig. 2 in terms of Cd concentration, the highest Cd concentration in the study was determined to be 9.31 mg Cd kg^−1^ with the 0% BC and 5 mg Cd kg^−1^ application. The highest Cd concentration in the 1% BC application was also determined with the 5 mg Cd kg^−1^ application (7.95 mg kg^−1^). In the study, the lowest Cd concentrations were determined in the control application, with 1.29 mg Cd kg^−1^ for the 0% BC application and 1.17 mg Cd kg^−1^ for the 1% BC application.

The highest N concentration was determined 3.90% N with the 0% BC and 2.5 mg Pb kg^−1^ application. This was followed by the 1% BC and 2.5 mg Pb kg^−1^ application with 3.59% N (Table 2). Similarly, the highest P concentration was also determined to be 0.30% P with the 0% BC and 2.5 mg Pb kg^−1^ application. Under 1% BC applications, the highest P concentration of 0.19% P was observed in the control application. Both N and P concentrations of the plant decreased under heavy metal applications with 1% BC. However, in the control treatments, N and P concentrations were 1.71% N and 0.13% P under 0% BC, respectively, whereas they increased to 3.15% N and 0.19% P under 1% BC, indicating a positive effect of BC application. In contrast to the N and P, the K concentration in the plant increased with Cd and Pb applications in both the 0% BC and 1% BC applications. The highest K concentration was determined to be 5.35% K with the 1% BC and 5 mg Pb kg^−1^ application, while the lowest concentration was found to be 3.61% K in the 0% BC and control applications. In the study, the K concentration of parsley grown under both Cd and Pb applications was found to be higher in the 1% BC application compared to the 0% BC application. This indicates that the interaction between BC and heavy metals is significant.

In the study, the Ca concentration in parsley plants showed a similar trend to the N and P concentrations (Table 3). Ca responses differed depending on the combined effects of BC and heavy metal applications. Under 0% BC application, all Cd and Pb applications increased Ca concentrations compared with the control application (1.39% Ca). However, under 1% BC applications, Ca concentrations decreased depending on the heavy metal treatments, except for the 5 mg Pb kg^−1^ application, indicating a BC × heavy metal interaction. The highest Ca concentration in the study was determined as 2.24% Ca under the 1% BC and 5 mg Pb kg^−1^ application. The Mg concentration in the plant decreased with Cd and Pb applications compared to the control in the 0% BC application. However, with the 1% BC application, the Mg concentration generally increased compared to the control, and the highest concentration of 0.96% Mg was observed with the 1% BC and 2.5 mg Pb kg^−1^ application. These results indicate that the effect of BC on Mg concentration depended on the type and level of heavy metal application. Overall, the study found that while the plant’s Ca concentration decreased with BC application, the Mg concentration increased with BC application in the presence of Cd and Pb. This indicates that the BC × heavy metal interaction was not significant for Ca concentration but was significant for Mg concentration.

When examining the micronutrient concentrations in parsley plants in Table 4, the highest Fe concentration was determined to be 674.6 mg Fe kg^−1^ with the 1% BC and control applications. In the study, under the 1% BC application, the Fe concentration of the plant was found to be higher, particularly in the Pb applications, compared to the 0% BC application. Zn concentrations also varied depending on treatment combinations. Under 0% BC applications, the highest Zn concentration was determined to be 80.6 mg Zn kg^−1^ with the 2.5 mg Pb kg^−1^ application, whereas under 1% BC applications, Zn concentrations were generally higher in Pb applications compared with Cd applications. The effect of BC on Zn concentrations differed among heavy metal treatments, indicating application specific responses. Mn concentrations ranged between 85.4 and 190.8 mg Mn kg^−1^ across applications. The highest Mn concentration, 190.8 mg Mn kg^−1^, was obtained with the 5 mg Pb kg^−1^ application. The highest Cu concentration, 17.8 mg Cu kg^−1^, was determined with the 0% BC and 2.5 mg Pb kg^−1^ application, while the lowest concentration, 5.3 mg Cu kg^−1^, was observed with the 1% BC and 5 mg Cd kg^−1^ application. When the research findings were evaluated as a whole in terms of the plant’s micronutrient concentrations, it was determined that with BC application, particularly under Pb application, Zn and Mn concentrations increased significantly compared to the control treatment. This indicates a positive interaction between BC and Pb. This study investigated changes in the CAT, POD, and APX enzyme activities by applying different concentrations of Cd and Pb to parsley plants. The effect of BC application on these changes was also evaluated. The analysis data is provided in Table 5.

**Table 5:** Effects of different doses of BC, Cd, and Pb applications on CAT, POD, and APX concentrations of parsley plants (EU g^−1^ fw).

Applications		CAT	POD	APX
		EU g^−1^ fw		
0% BC	Control	79.3 ± 3.20^a^*	0.079 ± 0.00^a^	0.62 ± 0.06^ab^
	2.5 mg Cd kg^−1^	49.8 ± 3.41^de^	0.031 ± 0.01^de^	0.46 ± 0.08^b–d^
	5 mg Cd kg^−1^	40.2 ± 2.83^f^	0.011 ± 0.01^f^	0.37 ± 0.09^cd^
	2.5 mg Pb kg^−1^	51.7 ± 1.98^c–e^	0.021 ± 0.00^ef^	0.39 ± 0.04^cd^
	5 mg Pb kg^−1^	46.4 ± 3.07^ef^	0.016 ± 0.00^ef^	0.16 ± 0.06^e^
**Average**		**53.5 B**	**0.03 B**	**0.40 B**
1% BC	Control	81.7 ± 4.89^a^	0.083 ± 0.00^a^	0.69 ± 0.08^a^
	2.5 mg Cd kg^−1^	67.1 ± 3.51^b^	0.045 ± 0.01^cd^	0.53 ± 0.03^a–c^
	5 mg Cd kg^−1^	57.8 ± 2.57^cd^	0.027 ± 0.01^e^	0.44 ± 0.05^b–d^
	2.5 mg Pb kg^−1^	60.7 ± 2.91^bc^	0.064 ± 0.01^b^	0.50 ± 0.08^b–d^
	5 mg Pb kg^−1^	54.8 ± 3.22^c–e^	0.048 ± 0.01^c^	0.34 ± 0.06^de^
**Average**		**64.4 A**	**0.05 A**	**0.50 A**

**Notes:**

*P* < 0.05.

Mean of three replicates and ± is a standard error.

*The letters used in the table indicate the statistical differences between the groups.

In the study, the effects of BC applications on the enzyme activities of parsley plants grown under Cd and Pb stress were investigated. In the study, the control groups in both 0% BC and 1% BC applications exhibited the highest activities of CAT, POD, and APX enzymes, with values of 79.3 EU g^−1^ fw, 81.7 EU g^−1^ fw, 0.079 EU g^−1^ fw (for 0% BC), and 0.083 EU g^−1^ fw, 0.62 EU g^−1^ fw, and 0.69 EU g^−1^ fw (for 1% BC), respectively. As the concentrations of Cd and Pb increased (from 2.5 to 5 mg kg^−1^), a pronounced decline in enzyme activities was observed, indicating a dose-dependent inhibitory effect of these metals on the plant’s antioxidant defense system. The lowest CAT and POD activities were determined in the 0% BC and 5 mg Cd kg^−1^ application, with values of 40.2 and 0.011 EU g^−1^ fw, respectively, while the lowest APX activity was determined in the 0% BC and 5 mg Pb kg^−1^ application, with a value of 0.16 EU g^−1^ fw. POD activity, in particular, exhibited the highest sensitivity to heavy metal applications. In the 5 mg Cd kg^−1^ and 5 mg Pb kg^−1^ applications, POD activity decreased by approximately 80–90% compared to the control group.

## Discussion

In the study, dry weight findings are significant in improving the growth and development of parsley plants in soils contaminated with Cd and Pb through BC application. Similarly, in a study where BC (0% and 2% w/w) and four different Cd doses (0, 2, 4, and 8 mg Cd kg^−1^) were applied to maize plants in the form of CdSO_4_, the highest dry weight was obtained from the 2% BC and 4 mg Cd kg^−1^ application 27.52 g pot^−1^. It was reported that while increasing Cd doses reduced dry weight under the 0% BC application, all Cd applications under the 2% BC application increased dry weight compared to the control (Demirbaş & Coşkan, 2019). In low-yielding soils of Nepal, four applications were applied to maize plants: (1) control without biochar (CK), (2) biochar (BC), (3) biochar + fertilizer (BC+M), and (4) urine-enriched biochar + fertilizer (BU+M). At the end of the study, it was reported that soil nutrient levels (N, P, and K) were significantly improved, and maize yield increased by 62% compared to the CK application with the application of BC (Pandit et al., 2024).

When the average Cd concentrations were examined, it was observed that the Cd concentration in parsley plants decreased by 14.4%, especially with the 1% BC application. However, the opposite situation was observed in the Pb application. With the 1% BC application, the average Pb concentration in parsley plants increased by 5.9%. It was determined that the Cd application increased Pb uptake. However, in the study, the Pb concentration was found to be 38.95 mg Pb kg^−1^ under the 0% BC and 5 mg Pb kg^−1^ application, while with the 1% BC application at the same dose, it decreased to 27.70 mg Pb kg^−1^. In the 2.5 mg Pb kg^−1^ application, the Pb concentration was found to be 29.85 mg Pb kg^−1^, while in the 5 mg Pb kg^−1^ application, it was determined to be 27.70 mg Pb kg^−1^. A study examining the adsorption kinetics and mechanisms for the removal of metal ions such as lead (Pb^2+^) and cadmium (Cd^2+^) using BC obtained from cattle manure reported that after the basic application, the adsorption capacity of BC for Pb^2+^ and Cd^2+^ increased. The highest adsorption capacities were found to be 175.53 mg g^−1^ for Pb^2+^ and 68.08 mg g^−1^ for Cd^2+^ (Chen et al., 2019). In a study where BC produced from sugarcane bagasse at 700 °C was applied to mine-contaminated soil at 1.5%, 3.0%, and 5.0% (w/w) rates to Jack bean (*Canavalia ensiformis*) and *Mucuna aterrima* plants, it was reported that BC application reduced the uptake of Cd, Pb, and Zn by the plants grown in the mine contaminated soil. The concentrations of these metals were decreased by 56%, 50%, and 54%, respectively (Puga et al., 2015). The study reported that the application of BC during soil remediation in mine-contaminated areas would reduce the potential toxic metal concentrations in plants. In a study investigating the potential of BC derived from various biomass sources such as apricot kernel (AK), pine sawdust (PS), rice husk (RH), wheat straw (WS), and reed stem (RS) to improve the yield, nutritional quality, and environmental sustainability of parsley plants, biochar-enriched iodine and selenium fertilizer (BISF) was also applied. At the end of the study, it was reported that the application of BISF significantly improved the main biometric parameters, with the weight of the parsley leaves being a critical indicator of the plant’s overall growth and biomass production. The leaf weight increased by 1.6 times compared to the control, reaching 326.5 g (Doszhanov et al., 2024). In addition, they highlighted the effectiveness of selenium and iodine-enriched BC in increasing the yield and nutritional quality of parsley plants, and reported that BC demonstrated a multifunctional role in environmental improvement. In this study, when the Cd and Pb concentrations in parsley plants were evaluated as a whole, it is believed that the application of BC could reduce the negative effects of these two important heavy metals, which can create stress in plants and potentially reduce their growth and development. In a similar study, Arsenov et al. (2021) investigated the presence of potential toxic elements (PTEs) such as As, Cu, Fe, Mn, Ni, and Cd in the soil with celery and parsley plants, and examined the plants’ physiological responses to different Cd exposures (control group without Cd, 3, and 6 µg g^−1^ dry soil Cd levels). They reported that with the increase in Cd levels, there was an increasing trend in the accumulation of As, Pb, and Cu in both species, with the levels of these elements exceeding safe limits, except for Cu.

In the study, the concentrations of the macronutrients N, P, and K were evaluated, in the 0% BC application, all Cd and Pb applications increased the N concentration in parsley plants. In comparison, in the 1% BC application, the N concentration in the plant generally decreased with Cd and Pb applications. Similar to the N concentration, in the 0% BC application, all Cd and Pb applications increased the P concentration in parsley plants. However, in the 1% BC application, the Cd and Pb applications decreased the P concentration of the plant. When the average N, P, and K concentrations were evaluated in the study, it was determined that the BC application did not affect the N and P concentrations (2.61% N and 0.15% P, respectively) but had a positive effect on the K concentration (5.17% K). The study found that while BC application did not statistically affect the plant’s Ca concentration, it significantly affected the Mg concentration (0.90% Mg). In a similar study, BC produced at different pyrolysis temperatures was applied to maize plants at various doses, and it was reported that Ca concentrations ranged between 0.15–0.48% Ca, while Mg concentrations ranged between 0.24–0.52% Mg (Tepecik, Kayıkçıoğlu & Kılıç, 2022).

When the data are evaluated in terms of microelement concentrations in the 0% BC application, the Fe concentration in the plant was higher with Cd application compared to the control, while it decreased with Pb application. In the 1% BC application, all Cd and Pb applications reduced the plant’s Fe concentration. In the 0% BC application, all Cd and Pb applications increased the plant’s Zn concentration. In comparison, in the 1% BC application, the Zn concentration decreased with Cd application compared to the control, but increased with Pb application. the Mn concentration in the plant decreased with all Cd and Pb applications compared to the control, except for the 2.5 mg Pb kg^−1^ application in the 0% BC application. In the 1% BC application, a lower Mn concentration was obtained with Cd application compared to the control, while an increase was observed with Pb application. In the study, the BC application did not affect the Cu concentration in the plant, and all applications had lower Cu concentrations compared to the control. When evaluating the micronutrient concentrations as a whole in the study, the 1% BC application affected the Fe, Zn, and Mn concentrations in parsley plants grown in Cd and Pb-contaminated soil (412.8 mg Fe kg^−1^, 50.2 mg Zn kg^−1^, and 140.6 mg Mn kg^−1^, respectively). However, it did not affect the Cu concentration, which was 11.1 mg Cu kg^−1^. In a study by Demirbaş & Coşkan (2019), where they applied 0% and 2% BC with 0, 2, 4, and 8 mg Cd kg^−1^ to maize plants, it was reported that the Fe, Mn, and Cu concentrations were higher with the 2% BC application (81.26 mg Fe kg^−1^, 52.98 mg Mn kg^−1^, and 7.05 mg Cu kg^−1^, respectively), while the Zn concentration was higher with the 0% BC application (18.29 mg Zn kg^−1^).

Determining the ability of plants to cope with Cd and Pb stress is essential for a comprehensive understanding of the mechanisms underlying heavy metal tolerance and toxicity. Heavy metals disrupt redox homeostasis by reducing antioxidant activity. Specifically, Cd has been shown to impair mitochondrial function by decreasing the activity of antioxidant enzymes (Koyama, Kamogashira & Yamasoba, 2024). In this study, the activities of antioxidant enzymes varied depending on the type and concentration of the applied heavy metal (Table 5). Furthermore, notable differences in antioxidant enzyme activities were observed in response to BC application. Cd and Pb applications generally resulted in a marked decrease in the activities of antioxidant enzymes, including CAT, POD, and APX. Both Cd and Pb were found to suppress antioxidant enzyme activities in parsley plants. The study findings demonstrate that the enzymatic antioxidant defense system in parsley plants is insufficient to mitigate the oxidative stress induced by heavy metals, due to the marked suppression of enzyme activities. These findings from the study are consistent with previous studies reporting that exposure to Cd and Pb disrupts cellular redox homeostasis and inhibits enzymatic antioxidant defenses in various plant species. Ulusu, Öztürk & Elmastaş (2017) applied different concentrations of CdCl₂ (0, 75, 150, and 300 μM) to the leaves of parsley (*Petroselinum hortense* L.) seedlings grown under greenhouse conditions for 3 months, to determine the antioxidant capacity and Cd accumulation in the plant. As a result, they found that the Cd level in parsley leaves increased in parallel with the rise in soil Cd contamination, while the total chlorophyll and carotenoid contents significantly decreased with increasing Cd concentration. In addition, they reported that the activity of the antioxidant enzyme SOD partially increased at 75 and 150 μM CdCl_2_ concentrations, but decreased at 300 μM CdCl_2_. In contrast, the activities of CAT and APX decreased in response to Cd application. These results confirm that Cd accumulation suppressed antioxidant enzyme activities in parsley leaves. Ak & Yücel (2011) investigated the effect of heavy metal stress on antioxidant enzyme levels in the wheat variety *Triticum aestivum* cv. Alpu. In their study, they applied different concentrations (100, 200, and 300 µM) of Cd, Pb, and Cd+Pb to the wheat plants and measured the activities of the SOD and CAT enzymes. As a result of the study, they found that heavy metals altered enzyme activities depending on their type and concentration. While Cd increased SOD and CAT enzyme activities compared to the control in a concentration-dependent manner, decreases were observed in these enzyme activities under Pb and Cd+Pb applications compared to the control. In contrast to the present study’s findings, Arsenov et al. (2021) investigated the physiological responses of celery and parsley plants to different levels of Cd exposure (control–no Cd added, 3 and 6 µg/g dry soil Cd). In their study, they found that the presence of Cd in the plants led to the activation of GPX (glutathione peroxidase**)**, CAT, APX, and GST (glutathione S-transferase), and that there was a strong positive correlation between Cd content and proline content, as well as APX, GPX, and CAT activities.

On the other hand, in the study, the 1% BC application led to improvements in all enzyme activities. The increased enzyme activity was associated with reduced heavy metal-induced suppression of antioxidant enzymes due to the BC application. However, enzyme activities remained lower than those in the control plants despite this increase. In particular, the BC application was observed to reduce Cd and Pb toxicity regarding CAT and POD activities. In addition, Cd suppressed CAT enzyme activity more than Pb. However, with the BC application, the increase in Cd application’s enzyme activity was greater than Pb. In contrast, POD activity was more strongly suppressed by increasing Pb concentration than by Cd. However, with the BC application, the increase in enzyme activity in Pb applications was greater than in Cd applications. For example, in the 0% BC and 5 mg Cd kg^−1^ application, CAT activity was 40.2 EU g^−1^ fw, whereas in the presence of 1% BC, it increased to 57.8 EU g^−1^ fw. Similarly, in the 0% BC and 5 mg Pb kg^−1^ application, POD activity was 0.016 EU g^−1^ fw, and with 1% BC application, it increased to 0.048 EU g^−1^ fw. In the study, BC application significantly increased CAT, POD, and APX enzyme activities by 20.4%, 66.7%, and 25.0%, respectively. These results indicate that BC helps maintain CAT, POD, and APX enzyme activities under heavy metal stress and supports the plant’s enzymatic antioxidant defense mechanism by activating it against oxidative damage. BC may reduce the bioavailability of heavy metals by binding them, thereby alleviating oxidative stress. Mirmohammadmakki et al. (2023) added untreated and phosphoric acid-treated almond shells at different concentrations to the soil of parsley plants as a biosorbent, and investigated the effect of the almond shells on reducing heavy metal (Pb, Cd, Ni, and As) accumulation in parsley. According to the findings, the uptake of the investigated metals depended on factors such as the initial concentration of the metals and the duration of the biosorbent’s presence in the soil. Heavy metal levels were reduced in all soil samples containing the biosorbent. The highest reduction in soil heavy metal content was recorded for Pb, followed by Cd. The highest decrease was observed in the samples containing biosorbent treated with phosphoric acid. Studies conducted on parsley leaves showed a strong correlation between the addition of biosorbent to the soil and the decrease in heavy metal content in the parsley. In addition, a strong relationship was found between the presence of biosorbent in the soil and the amount of residual heavy metals in parsley. The results showed that as the amount of biosorbent in the soil increased, the heavy metal content in parsley decreased accordingly. In a study investigating the potential of BC and BISF (biochar-enriched iodine and selenium fertilizer) derived from various biomass sources such as apricot kernel (AK), pine sawdust (PS), rice husk (RH), wheat straw (WS), and reed stem (RS) to improve the yield, nutritional quality, and environmental sustainability of parsley plants, BISF application was reported to increase antioxidant content—as measured by ascorbic acid, polyphenols, and antioxidant activity—by 1.56, 1.27, and 1.50 times, respectively (Doszhanov et al., 2024).

The study results support that parsley plants exhibit strong antioxidant properties and high tolerance to heavy metals in the presence of BC. However, this should not lead to the mistaken conclusion that edible plants such as parsley grown in heavy metal-contaminated environments are safe for consumption. Studies indicate that consuming plants contaminated with heavy metals can adversely affect human health (Guo, Zhang & Wang, 2019; Rai et al., 2019; Maleki & Zarasvand, 2008).

## Conclusion

Heavy metal pollution has become an increasingly worsening problem today. This situation has led to a growing focus on research concerning the phytotoxicity of heavy metals and the mechanisms that plants use to cope with these harmful effects. In particular, the potential contamination of edible medicinal plants with heavy metals is an increasingly growing concern. Medicinal plants are known for their use in improving human health; however, to ensure maximum benefit for consumers and to prevent unwanted effects, more detailed information about their chemical composition is required (Arsenov et al., 2021; Kohzadi et al., 2019; Qinsong & Guoxin, 2000).

Pb and Cd are among the most abundant and toxic heavy metals (Kavvadias et al., 2012). Heavy metals such as Cd and Pb are non-essential elements for plants. When they accumulate in high amounts, these metals negatively affect the uptake and transport of essential elements, disrupt plant metabolism, and adversely affect growth and reproduction (Qinsong & Guoxin, 2000; Cheng, 2003). In the study, the plant’s green dry weight (plant biomass) decreased under heavy metal application in 0% and 1% BC applications. However, BC application increased dry weight by 17.8% compared to parsley plants without BC, promoting greater growth and development. In addition, with BC applications, the Cd concentration in the plant decreased by 14.4%, while the concentrations of K, Mg, Fe, Zn, and Mn in parsley increased by 15.9%, 5.9%, 69.3%, 5.5%, and 17.6%, respectively.

Due to its low cost and frequent use in various dishes and medicinal purposes, parsley is significant among plant-based products that may accumulate heavy metals. Therefore, if commonly consumed plants like parsley contain heavy metals, it is likely that humans’ daily intake of heavy metals may exceed safe limits, leading to an increased risk of health problems. In this context, determining heavy metal accumulation and antioxidant enzyme activities in green leafy plants with high antioxidant content is of great importance. Studies have indicated that in industrialized regions, approximately 60–80% of heavy metals in the human body, particularly Cd, are taken in through food consumption (Piekut et al., 2018). In this study, the applied doses of Cd and Pb suppressed and reduced the activities of the antioxidant enzymes CAT, POD, and APX. The BC application was effective in reducing the oxidative stress caused by Cd and Pb, particularly helping to maintain higher levels of CAT and POD activities. With the BC application, increases of 20.4%, 66.7%, and 25.0% were observed in CAT, POD, and APX enzyme activities, respectively. The results of this study indicate that BC could be used as a potential remediation agent against heavy metal pollution in agricultural areas.

By applying BC to the soil, Cd and Pb toxicity can be reduced, and crop productivity can be improved. Through further studies, the effects of different doses of BC applications and the long-term impacts of heavy metals can be investigated. These findings are important for understanding plant defense mechanisms against heavy metal stress and developing sustainable solutions to improve agricultural soils.

## Supplemental Information

10.7717/peerj.21144/supp-1 Supplemental Information 1 Raw Data. The complete raw data used for all statistical analyses including treatment groups, replicate values, and measured parameters.

## Additional Information and Declarations

### Data Availability

The following information was supplied regarding data availability:

The raw data is available in the Supplemental File.
